# Dual roles of the adenosine A2a receptor in autoimmune neuroinflammation

**DOI:** 10.1186/s12974-016-0512-z

**Published:** 2016-02-26

**Authors:** J. Ingwersen, B. Wingerath, J. Graf, K. Lepka, M. Hofrichter, F. Schröter, F. Wedekind, A. Bauer, J. Schrader, H.-P. Hartung, T. Prozorovski, O. Aktas

**Affiliations:** Department of Neurology, Medical Faculty, Heinrich-Heine-University Düsseldorf, Moorenstr. 5, 40225 Düsseldorf, Germany; Institute of Neuroscience and Medicine, INM-2, Research Center Jülich, Leo-Brandt-Str., 52425 Jülich, Germany; Cardiovascular Physiology, Medical Faculty, Heinrich-Heine-University Düsseldorf, Universitätsstr. 1, 40225 Düsseldorf, Germany; Current address: Institute for Stem Cell Research and Regenerative Medicine, Medical Faculty, HeinrichHeine University, Moorenstrasse 5, 40225 Düsseldorf, Germany

**Keywords:** Adenosine, Multiple sclerosis, Experimental autoimmune encephalomyelitis

## Abstract

**Background:**

Conditions of inflammatory tissue distress are associated with high extracellular levels of adenosine, due to increased adenosine triphosphate (ATP) degradation upon cellular stress or the release of extracellular ATP upon cell death, which can be degraded to adenosine by membrane-bound ecto-enzymes like CD39 and CD73. Adenosine is recognised to mediate anti-inflammatory effects via the adenosine A2a receptor (A2aR), as shown in experimental models of arthritis. Here, using pharmacological interventions and genetic inactivation, we investigated the roles of A2aR in experimental autoimmune encephalomyelitis (EAE), an animal model of multiple sclerosis (MS).

**Methods:**

We used two independent mouse EAE variants, i.e. active immunization in C57BL/6 with myelin oligodendrocyte glycoprotein (MOG)_35-55_ or transfer-EAE by proteolipid protein (PLP)_139-155_-stimulated T lymphocytes and EAE in mice treated with A2aR-agonist CGS21680 at different stages of disease course and in mice lacking A2aR (A2aR^−/−^) compared to direct wild-type littermates. In EAE, we analysed myelin-specific proliferation and cytokine synthesis ex vivo, as well as inflammation and demyelination by immunohistochemistry. In vitro, we investigated the effect of A2aR on migration of CD4^+^ T cells, macrophages and microglia, as well as the impact of A2aR on phagocytosis of macrophages and microglia. Statistical tests were Mann-Whitney *U* and Student’s *t* test.

**Results:**

We found an upregulation of A2aR in the central nervous system (CNS) in EAE, predominantly detected on T cells and macrophages/microglia within the inflamed tissue. Preventive EAE treatment with A2aR-specific agonist inhibited myelin-specific T cell proliferation ex vivo and ameliorated disease, while application of the same agonist after disease onset exacerbated non-remitting EAE progression and resulted in more severe tissue destruction. Accordingly, A2aR-deficient mice showed accelerated and exacerbated disease manifestation with increased frequencies of IFN-γ-, IL-17- and GM-CSF-producing CD4^+^ T helper cells and higher numbers of inflammatory lesions in the early stage. However, EAE quickly ameliorated and myelin debris accumulation was lower in A2aR^−/−^ mice. In vitro, activation of A2aR inhibited phagocytosis of myelin by macrophages and primary microglia as well as migration of CD4^+^ T cells, macrophages and primary microglia.

**Conclusions:**

A2aR activation exerts a complex pattern in chronic autoimmune neurodegeneration: while providing anti-inflammatory effects on T cells and thus protection at early stages, A2aR seems to play a detrimental role during later stages of disease and may thus contribute to sustained tissue damage within the inflamed CNS.

**Electronic supplementary material:**

The online version of this article (doi:10.1186/s12974-016-0512-z) contains supplementary material, which is available to authorized users.

## Background

The purine nucleoside adenosine is a modulatory messenger that is implicated in many physiological and pathological functions of both the immune system and the central nervous system (CNS). Conditions of tissue injury are associated with high extracellular levels of adenosine. The source of adenosine can either be intracellular adenosine triphosphate (ATP) degradation upon cellular stress, particularly under high energy demand, and subsequent transportation to the extracellular space via specialised nucleoside transporters or the appearance of large amounts of extracellular ATP due to tissue damage and cell death, which can be degraded to adenosine by membrane-bound ecto-enzymes like CD39 and CD73 [1]. Enhanced extracellular concentrations of adenosine can be considered as a general damage signal [2], contributing to a ‘damage-associated molecular pattern (DAMP)’ [3].

Among the four G-protein-coupled receptors activated by adenosine, adenosine receptor 2a (A2aR) is responsible for most of the known immunoregulatory effects of adenosine in the immune system, preventing overly exuberant immune responses that can lead to collateral tissue damage [4]. A2aR activation was shown to confer beneficial anti-inflammatory effects in models of ischemic or traumatic tissue damage in different organ systems, among them the liver, kidney, heart, skin and lung [5–10]. Moreover, regarding experimental autoimmunity in vivo, several reports have described a regulatory role for A2aR in collagen-induced arthritis [11] or a transgenic mouse model of T cell tolerance [12]. In the CNS, however, it was suggested that A2aR may enhance tissue injury, regardless of the underlying damage model: genetic or pharmacological inactivation of A2aR led to favourable outcomes in models of stroke [13–15], traumatic cortical brain injury [16, 17] and spinal cord damage [18, 19].

Multiple sclerosis (MS) is the most common chronic inflammatory disease of the CNS in Western countries characterised by demyelination, neuronal damage and glial scaring [20, 21]. Elevated adenosine levels have been detected in cerebrospinal fluid of MS patients [22, 23]. Since A2aR is expressed in both immune and primary CNS cells targeting the receptor may develop into a promising treatment strategy for MS. However, whether activation or blockade of the receptor will confer neuroprotective effects is hard to predict. In experimental autoimmune encephalomyelitis (EAE), an animal model of MS, the A2aR antagonist SCH58261 was shown to protect from the disease, and genetic ablation of CD73, a molecule that generates adenosine, resulted in milder EAE course [24]. Follow-up studies indicated that both CNS- and immune cell-derived A2aR regulate EAE [25], and that loss of A2aR exacerbates EAE pathology [26]. The exact molecular mechanisms and the specification of the involved cell type remained, however, unknown.

We here demonstrate that modulation of A2aR in the course of EAE depends on the disease phase and the affected tissue compartment, comprising differential A2aR-dependent regulation of invading T lymphocytes and macrophages/microglia.

## Methods

### Experimental autoimmune encephalomyelitis

Six- to eight-week-old A2aR-deficient mice generated by Chen et al*.* and bred on a C57BL/6 background [13] or wild-type littermates of the A2aR-deficient mouse strain were immunised subcutaneously with 200 μg of recombinant myelin oligodendrocyte glycoprotein (MOG_35-55_; Pepceuticals) and 800 μg mycobacterium tuberculosis (H37RA; Difco) emulsified in 100 μl PBS and 100 μl complete Freund’s adjuvant (CFA; Difco) per mouse, as previously described [27]. Pertussis toxin (PTX; 200 ng; List Biological Laboratories) was administered intraperitoneally on the day of immunization and on day 2. For transfer experiments, donor mice were immunised as described, but without the use of PTX and were sacrificed at day 10 post immunization. For EAE experiments without investigation of A2aR deficiency, 6–8-week-old wild-type female mice with C57BL/6 background (Janvier) were used. Spleen and lymph node cells were isolated and cultured in the presence of 10 μg/ml MOG for 4 days. Ten million cells were transferred i.p. into recipient mice. For expression pattern analysis in adoptive transfer, EAE donors and recipients were 6–8-week-old female SJL/J mice (Janvier) and peptide for induction was proteolipid protein (PLP_139–151_; Pepceuticals), as previously described [28]. Mice were scored for EAE as follows: 0, no disease; 1, tail weakness; 2, paraparesis; 3, paraplegia; 4, paraplegia with forelimb weakness or paralysis; 5, moribund or dead animals, and intermediate steps, if applicable [29]. Mean clinical scores at separate days were calculated by adding scores of individual mice and dividing by the number of mice in each group. Statistical analysis was performed by the non-parametric Mann-Whitney *U* test. All mice were housed specific pathogen-free, at a dark/light cycle of 12 h and stable temperature of 22–24 °C and had unlimited access to food and water. All procedures were conducted according to protocols approved by the local animal welfare committee and comply with the ARRIVE criteria [30].

### Real-time PCR

RNA isolation of tissue samples was performed using TRIzol® solution according to the manufacturer’s instructions (Invitrogen). Purity and amount of RNA were measured with Nanodrop 2000 (Thermo Scientific). cDNA was synthesised with TaqMan reverse transcription reagents (Applied Biosystems). qRT-PCR was performed with Power SYBRGreen fluorescent dye (Applied Biosystems) or TaqMan probe (5′Fam, 3′TAMRA) and the 7500 Real-Time PCR System (Applied Biosystems). Primers were designed using Primer Express software (Applied Biosystems). Each measurement was performed in duplicates. To determine the expression of A2aR mRNA in microglial or macrophage cultures, cells were treated with 50 μg/ml myelin for the indicated period times. For visualisation of PCR products, PCR reactions (after 35 cycles) were loaded on 2 % agarose gel and electrophoresis was performed. Images were captured by a Biometra Image system. For all samples, glyceraldehyde 3-phosphate dehydrogenase (GAPDH) was used as housekeeping gene. *P* < 0.05 was determined to be statistically significant by Student’s *t* test.

### Western blot

For protein extractions, tissue or cell pellets were dissolved in RIPA buffer containing 10 mM protease inhibitor cocktail (Roche). Proteins (25 μg/μl) were separated by gel electrophoresis on 8 % SDS-PAGE (Invitrogen) due to their molecular weight. Protein lysate was diluted in RNAse-free water to a concentration of ~5–10 μg/μl, and 4× loading buffer (LI-COR) was added. Blotting was performed using iBlot dry blotting system (Invitrogen) for 10 min at ~20 V. The membranes were blocked for 1 h at RT in 5 % skimmed milk diluted in 0.05 % PBS-Tween (blocking buffer). Afterwards, the membrane was incubated with primary antibody (anti-A2aR antibody, AAR-002; Alomone Labs) diluted in blocking buffer overnight at 4 °C. The next day, the membrane was washed three times for 5 min in 0.05 % PBS-Tween and later incubated with secondary antibody (LI-Cor IRDye conjugated) diluted in an appropriate amount of 0.05 % PBS-Tween for 1 h at RT under photosensitive conditions. Odyssey Infrared Imaging System detecting at 680 and 800 nm (LI-COR) was used to detect the infrared signals. For quantitative analysis, protein expression was normalised to the expression level of the housekeeping protein (β-actin).

### Proliferation assay

Lymph nodes and spleens were mashed through a cell strainer and lysed in ammonium chloride lysis buffer. Lymph node cells and splenocytes were cultured in the presence of recombinant MOG_35-55_-peptide or anti-CD3 antibody (clone 145-2C11, 1 μg/ml; eBioscience) as positive control. After 54 h, 0.5 μCi ^3^H-thymidine was added to each well. Eighteen hours later, cells were harvested and the proliferation rate was measured as counts per minute (cpm) in a β-counter (Wallac). *P* < 0.05 was determined to be statistically significant by Student’s *t* test.

### Intracellular cytokine staining

Spleen cells were harvested and CD4^+^ T cells isolated using MACS® CD4^+^ T cell isolation kit II mouse (Miltenyi Biotec) were restimulated with 10 μg/ml MOG_35-55_ and irradiated antigen-presenting cells (APC) (ratio 1:5). Intracellular cytokine staining was performed using Leukocyte Activation Cocktail and Fix/Perm buffer according to the manufacturer’s instructions (Becton Dickinson). Antibodies against CD4, interferon (IFN)-γ, interleukin (IL)-17A and granulocyte macrophage colony-stimulating factor (GM-CSF) were used (all eBioscience). Data acquisition and analysis were performed with a FACSCanto II and FACSCalibur (Becton Dickinson) and FACSDiva (Becton Dickinson) and FlowJo software (Tree Star). *P* < 0.05 was determined to be statistically significant by Student’s *t* test.

### T cell migration assay

Cell migration assay was performed using a 96-well plate based chemotaxis system (Neuroprobe, Gaithersburg, MD), as described previously [27], in three independent experiments. CD4^+^ cells were isolated from mouse spleen cells using magnetic bead negative selection (Stemcell® Mouse CD4^+^ T Cell Enrichment Kit), were pre-activated with anti-CD3 and anti-CD28 antibodies (eBioscience) for 3 h in RPMI medium containing 10 % fetal calf serum and resuspended at 1 × 10^7^ cells/ml. Lower chambers were loaded with 29 μl of the chemokine CXCL12 in the indicated concentration (ranging from 1 μg/ml to 0.01 ng/ml; ImmunoTools, Friesoythe, Germany) and overlaid with a 5-μm pore-size filter membrane. The cells (20 μl, i.e. 2 × 10^5^ cells) were loaded on the hydrophobic surface surrounding each well on the membrane (upper chamber). CGS21680 was added in different concentrations in order to assess its effect on migration (upper and lower chambers always containing the same CGS21680 concentration). After 3 h of incubation, cells in the lower chamber were transferred to a 96-well plate and counted automatically using a Becton Dickinson FACSCanto II with a 96-well high throughput sampler (HTS) device.

### Immunohistochemistry

Mice were anaesthetised with isoflurane and cardially perfused with saline solution. The brain and spinal cord were isolated and fixed in 4 % paraformaldehyde and dehydrated in a 30 % sucrose solution. Specimens were then frozen in Tissue-Tek® (Sakura Fintek), stored at −80 °C and subsequently cut with a cryostat (Leica). The slices were then permeabilised with 0.5 % Triton and blocked using 5 % normal goat serum (Invitrogen) and 1 % bovine serum albumin. The primary antibodies were incubated overnight at +4 °C and the secondary antibody for 1 h at room temperature. The following primary antibodies were used: mouse anti-A2aR (1:200; Santa Cruz), guinea pig anti-GFAP (1:500; SySy); rat anti-MBP (1:500; Millipore); rat anti-CD3 (1:200; Serotec) and rabbit anti IBA-1 (1:400; Wako). The stainings were visualised using secondary antibodies conjugated to fluorophores (Cy2, Cy3, Cy5; Millipore). After washing, Hoechst dye 33258 (Molecular Probes) was used to counterstain nuclei. Slides were analysed on an Olympus BX51 microscope and Photoshop 5.0 software (Adobe).

### Oil Red O lipid staining

Specimens were frozen in Tissue-Tek® as described above. After drying the slices and removing the Tissue-Tek®, the contour of the tissue was traced with a Dako Pen. The sections were incubated in 70 μl of 100 % propylene glycol for 2 min and then stained with 70 μl Oil Red O solution (Abcam, Cambridge, UK) overnight at room temperature in a humidity chamber on a shaker. Next, sections were differentiated for 1 min in a mixture of 85 % propylene glycol in distilled water, rinsed in two changes of distilled water, then stained with 70 μl haematoxylin for 2 min and rinsed thoroughly in distilled water. Between all the steps, the liquids were removed with an aspiration system.

### Primary microglia cultures

Primary microglia were isolated from P5–P7 mouse cortex using MACS CD11b (Microglia) Microbeads kit according to the manufacturer’s protocols (Miltenyi Biotec). Cells were plated on poly-ornithine-coated plates (Sigma-Aldrich) and expanded in the presence of 100 U/ml M-CSF (ImmunoTools, Friesoythe, Germany) in DMEM media containing 10 % heat-inactivated fetal calve serum (FCS). For experiments, cells were plated in 6- or 24-well plates overnight and treatment has been performed on the next day.

### Proliferation of microglia

CD11b sorted cells were placed on poly-L-ornithine-coated coverslips (20.000 cells per slip) and incubated in the presence of 100 U/ml M-CSF. After 24 h of treatment with 250 nM CGS21680 or vehicle, cells were fixed with 4 % PFA and stained with rabbit anti-phosphorylated histone H3 (Se10) (1:1000; Abcam) for analysis of mitotic cells. Hoechst dye 33258 (Molecular Probes) was used to counterstain nuclei. Mean number with SEM of mitoses per visual field (three independent cultures) was used to calculate the percentage of mitotic cells.

### Bone marrow-derived macrophages

Bone marrow-derived macrophages (BMDM) were isolated using standard methods [31]. In brief, bone marrow cells were isolated from femurs and tibiae of female C57BL/6 mice, and 4 × 10^5^ cells were cultured in 10 ml RPMI medium supplemented with 100 U/ml M-CSF (ImmunoTools, Friesoythe, Germany) and 10 % heat-inactivated FCS in a 37 °C humidified incubator (5 % CO_2_). Medium was changed on day 3. On day 7, adherent cells were harvested using a cellstripper, washed, counted and used for experiments.

### Phagocytosis assays

For myelin phagocytosis, homologous myelin fraction was obtained from adult mouse brains according to Norton and Podulso [32]. Purified lipoproteins were labelled with Alexa Fluor 488 (protein labelling kit; Molecular Probes). Phagocytosis was analysed in primary microglial cultures and BMDM. Briefly, 50.000 cells were allowed to adhere on poly-ornithine-coated 24-well plates for 16 h. Cells were then pre-treated for 1 h with (250 nM) concentrations of CGS21680 or vehicle. Alexa 488-labelled myelin (5 μg/ml) or 4 μl of a 2.5 % aqueous suspension of fluorescent microspheres (carboxylate-modified polystyrene beads; L4530; Sigma-Aldrich, Germany) were added to culture at 37 °C for 6 h along with agonist or vehicle. The cells were washed five times with DMEM media to remove dish- or cell surface-bound residual fluorescent beads. Cells were examined using the GENios Pro plate reader (Tecan) at 485 nm excitation/535 nm emission (Alexa 488). Mean number with SEM of fluorescent intensity out of eight experiments (two independent cultures) was performed to calculate the phagocytic capacity of macrophages, and 4–6 experiments (derived from two independent cultures) were performed for analysis of microglial phagocytosis. Results were normalised to vehicle-treated cultures. *P* < 0.05 was determined to be statistically significant by Student’s *t* test.

### Migration analysis

Microglia/macrophages were suspended in standard medium in the presence of 250 nM CGS21680 or vehicle, and 30.000 cells (150 μl) were added to the upper well of each 8 μm diameter holes Transwell™ insert (Ibidi). After 1 h, chemokines IP10 (100 ng/ml; ImmunoTools) and MIG (100 ng/ml; ImmunoTools) were added to the lower well containing 400 μl of media. Cells were incubated for 24 h (37 °C, 5 % CO_2_). The cell-bearing filters were fixed in 4 % PFA for 15 min, rinsed with PBS, and the remaining microglia on the upper side of each filter were removed with a Q-tip. The filters were stained with 0.4 % trypan blue stain (Invitrogen), and the number of cells that had migrated to the underside was analysed (five random fields/filter) at ×20 magnification using an Olympus BX51 microscope and Photoshop 5.0 software (Adobe). Mean number with SEM of migrated cells out of four experiments (derived from two independent cultures) was performed to calculate the migration capacity. Results were normalised to vehicle-treated cultures. *P* < 0.05 was determined to be statistically significant by Student’s *t* test.

## Results

### Upregulation of A2aR expression in the inflamed brain during EAE

**Fig. 1:**
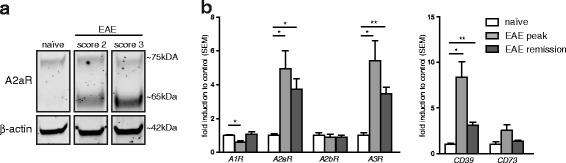
Expression of A2a receptors in autoimmune demyelination. **a** Expression of A2aR in naïve and active EAE (in C57BL/6 mouse strain) the spinal cord tissue. The protein A2aR has a molecular weight of approximately 42 kDA; the Western blotting band at ~65–70 kDa that we detected has been described for A2aR in the brain tissue before and is probably due to glycosylation [54, 55]. Use of a blocking peptide to the antibody against A2aR demonstrated specificity (see Additional file 1: Figure S1). **b** Expression of adenosine receptors and adenosine-generating ecto-enzymes in the spinal cord tissue in the course of adoptive transfer EAE (in SJL/J mouse strain). *p < 0.05, **p < 0.01

Virtually all immune cells express A2aR (reviewed in [33]). Regarding the CNS, A2aR is found in striatal neurons and in activated microglia [34, 35]. To identify the pattern of A2aR expression upon neuroinflammation, we induced EAE by active immunization of wild-type C57BL/6 mice with MOG_35-55_ and were able to detect a marked and sustained upregulation of A2aR expression within the spinal cord (Fig. 1a). We were able to confirm this finding in an independent EAE model in the SJL mouse strain, i.e. adoptive transfer EAE to avoid possible confounders associated with active immunization process. Here, an upregulation of messenger RNA (mRNA) level of the adenosine-generating ecto-enzymes CD39 and CD73, as well as of A2aR and A3R in the spinal cord tissue of EAE animals, was observed at the peak of the disease (day 12 post transfer, mean clinical score 3.5; *n* = 4; Fig. 1b). These changes in adenosine signaling were associated with downregulation of A1R, indicating the activation of a specific pattern of adenosine signaling. During chronic phase of the disease (day 50, mean clinical score 2; *n* = 4), the mRNA levels of most targets returned to basal level, while A2aR and A3R remained upregulated (Fig. 1b). These results indicate that disease severity and progression correlate with A2aR induction during the evolution of chronic myelin-specific neuroinflammation in the CNS.

### Opposing effects of pharmacological A2aR activation in early and late EAE

**Fig. 2:**
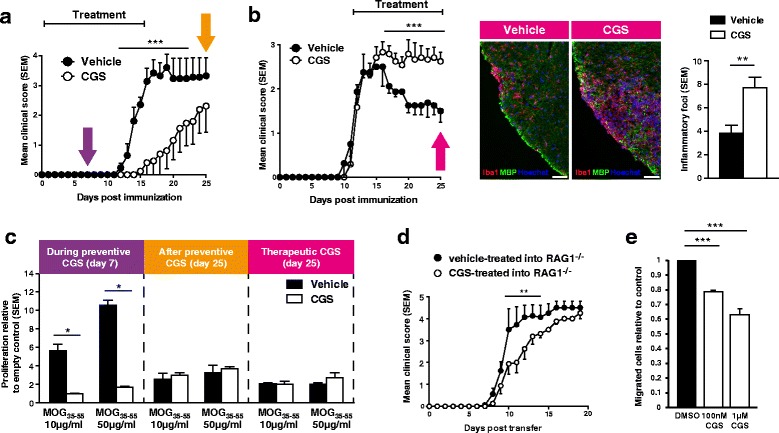
Effects of pharmacological A2aR activation in EAE. **a** Treatment with the A2aR-agonist CGS21680 (0.1 mg/kg) in the early phase of EAE leads to a prevention of disease symptoms (*n* = 8). *Coloured arrows* indicate the time points of ex vivo analyses. **b** *Left*: treatment with CGS21680 (0.1 mg/kg) in established disease (day 12 post immunization) leads to disease exacerbation (*n* = 5). *Middle*: histological analysis of CGS21680-treated EAE shows exacerbated Iba1+ accumulation in the spinal cord tissue (scale bars = 50 μm). *Right*: quantification of number of inflammatory foci at day 25 post immunization (*n* = 5). **c** Spleen cells isolated from vehicle-treated mice at day 7 in early-treatment paradigm (see *purple arrow* in Fig. 2a) show a higher antigen-specific proliferation capacity than cells isolated from CGS21680-treated mice. Cells isolated from late-stage early-treatment paradigm (*orange arrow* in Fig. 2a) and late-treatment paradigm (*pink arrow* in Fig. 2b) do not show differences in antigen-specific proliferation (*n* = 5). **d** T cells were transferred from EAE mice treated with CGS21680 or vehicle into naïve, non-immunised, non-treated RAG1-deficient mice (*n* = 4). **e** Analysis of migratory capacity towards CXCL12 in the presence or absence of CGS21680 of CD4^+^ T cells isolated from healthy mice using a Transwell chemotaxis system (data from three independent experiments). *p < 0.05, **p < 0.01, ***p < 0.001

We first aimed at an A2aR activation in pre-onset and early EAE and started the treatment with the A2aR-specific agonist CGS21680, known for its selectivity at this receptor [32] and its capacity to cross the blood-brain barrier [36–39], on the day of immunization (Fig. 2a). CGS21680 treatment (daily i.p. injections of 0.1 mg/kg) markedly delayed the onset of disease symptoms, and upon cessation of the treatment, the disease level closed in to that of vehicle-treated animals. Unexpectedly, in contrast to this preventive approach, treatment with the A2aR agonist CGS21680 starting at the peak of the disease (day 12 post immunization) had a detrimental effect on EAE clinical phenotype (Fig. 2b). CGS21680-treated mice were characterised by chronic non-remitting progressive disease course with severe neurological symptoms (mean score 3) as compared to the corresponding vehicle group. In line, an independent EAE run showed that continuous treatment with CGS21680 results in a lower disease score in the beginning of EAE (days 12–18) and an increased disease score at later EAE stage, i.e. after day 30 (Additional file 1: Figure S2). Immunohistochemical analysis correlated this clinical effect with increased numbers of inflammatory foci in the spinal cords of therapeutically CGS21680-treated mice. In order to elucidate the mechanisms behind this completely contrary effect of the same substance, we next analysed the T cell response in the different stages (Fig. 2c): in animals treated with CGS21680 beginning at immunization (i.e. during the development of the myelin-specific immune response in the peripheral immune system, see purple arrow in Fig. 2a), a marked difference in T cell response to MOG_35-55_ could be observed at day 7, pointing to an effect of A2aR on T cells in vivo. This effect on T cell proliferation, however, was non-existent after cessation of CGS21680 treatment (day 25 in preventive treatment, see orange arrow in Fig. 2a) and also non-existent in late-stage treatment (day 25 in therapeutic treatment, see pink arrow in Fig. 2b). To address the question whether T cells are the cells affected by CGS21680 and responsible for the early phenotype, we used adoptive transfer EAE. We transferred T cells from MOG_35-55_-immunised animals that had been treated with CGS21680 or vehicle for 7 days to naïve recombination-activating gene (RAG)1^−/−^ mice, lacking both T and B cells and thus exhibiting a high susceptibility to transfer EAE. Adoptive transfer from CGS21680-treated animals showed a milder onset of the disease (Fig. 2d). Additionally, CD4^+^ cells isolated from healthy animals were inhibited by CGS21680 treatment in their capacity to migrate towards a chemotactic stimulus (Fig. 2e and Additional file 1: Figure S3). Taken together, these results point to a key role of A2aR in the control of T cell activation and CNS invasion in the initiation phase of EAE. However, the data also indicate that A2aR activation in later-stage EAE may result in persistence of clinical deficits.

### EAE in A2aR^−/−^ mice shows a complex disease course

**Fig. 3:**
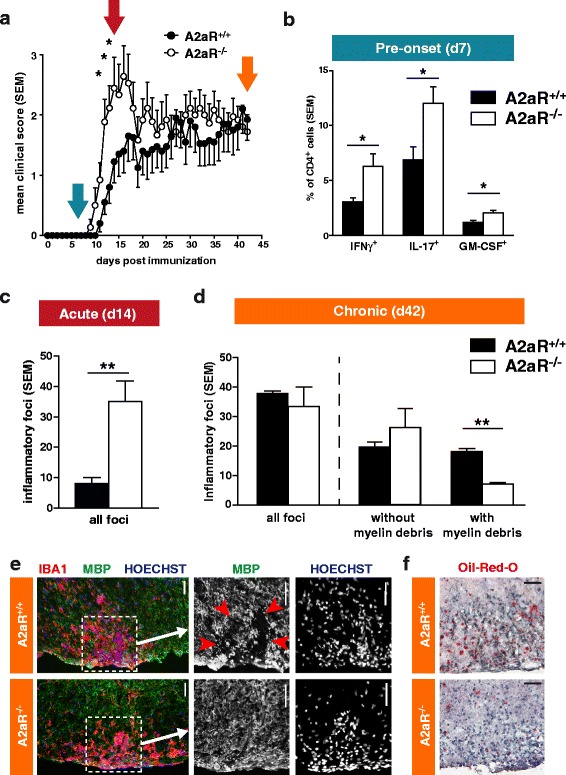
EAE in A2aR-deficient mice. **a** EAE was induced in A2aR^−/−^ and wild-type littermate C57BL/6 mice (*n* = 9). *Coloured arrows* indicate the time points of ex vivo analyses. **b** Ex vivo analysis of splenic CD4^+^ cells from A2aR^−/−^ and wild-type animals at day 7 post immunization with MOG_35-55_ peptide (see *blue arrow* in Fig. 3a). Cells were cultured in the presence of the antigen and irradiated APC for 5 days and then analysed for their cytokine production by flow cytometry (*n* = 5). **c** Quantification of inflammatory foci in the spinal cord at acute phase of EAE (day 14, see *red arrow* in Fig. 3a). **d** Quantification of inflammatory foci in the spinal cord and association with myelin debris at chronic phase of EAE (day 42, see *orange arrow* in Fig. 3a). *Bars on the left* show all foci, *bars on the middle and right* show the same foci differentiated into foci with and without myelin debris. **e** Representative images of histological analysis of the spinal cord damage in chronic phase. The images on the right correspond to a magnification of the image on the left (indicated by *dotted line and arrow*). *Red arrowheads* indicate the area with disruption of myelin tissue. Scale bars = 50 μm. **f** Oil red O lipid stain visualisation of myelin debris in spinal cord lesions (day 42; see also Additional file 1: Figure S5). Scale bars = 100 μm. *p < 0.05, **p < 0.01

Previous studies could show that in A2aR-deficient mice EAE course is quite unusual, with a marked increase in early A2aR-KO EAE and a recovery in these mice to the clinical level of wild-type (WT) EAE. We induced EAE by active immunization with MOG_35-55_ emulsified in CFA in C57BL/6 mice deficient for a functional adenosine-2a-receptor (hereafter A2aR^−/−^) and direct A2aR^+/+^ wild-type littermates (Fig. 3a). A2aR^−/−^ mice develop normally, and no morphological abnormalities have been observed [13]. As expected and in accordance with previous studies, A2aR^−/−^ mice showed an earlier onset and a stronger disease peak in the first days of clinical symptoms (Fig. 3a). In order to link this observation to underlying immunological mechanisms, we isolated CD4^+^ cells from MOG_35-55_-immunised animals just before the onset of clinical signs (on day 7, blue arrow) and restimulated them in vitro with MOG_35-55_ peptide. We observed increased numbers of IFN-γ-, IL-17- and GM-CSF-producing cells, key players in EAE development [40, 41], in CD4^+^ T cells isolated from A2aR^−/−^ mice as compared to their wild-type counterparts (Fig. 3b). In line, we found that naïve wild-type CD4^+^ T cells showed a markedly decreased antigen-independent proliferation (T cell receptor (TCR)-CD40L coactivation with anti-CD3 and anti-CD28) upon the non-receptor-specific adenosine analog 5′-*n*-ethylcarboxamidoadenosine (NECA) in vitro. This effect was only marginal in A2aR^−/−^ T cells, indicating that A2aR is the primary target of adenosine on T cells (see Additional file 1: Figure S4).

Following this early disease exacerbation, however, A2aR^−/−^ mice quickly recovered to a disease level similar to that of wild-type animals and after a follow-up of 6 weeks post immunization, no difference in clinical signs could be observed (Fig. 3a). In order to characterise the differences seen in early and late phases of EAE, we examined the CNS tissue histologically. In line with the early increased disease severity, we observed higher numbers of inflammatory foci in the spinal cord and brain of A2aR^−/−^ animals on day 14 (Fig. 3c and red arrow in Fig. 3a). The total number of inflammatory lesions was similar after the follow-up to day 42 (Fig. 3d and orange arrow in Fig. 3a). However, the number of inflammatory lesions that exhibited a myelin pattern suggestive of destruction was higher in the wild-type group. Figure 3e shows representative images from day 42 EAE of a lesion in A2aR^−/−^ without myelin damage and a lesion in A2aR^+/+^ with such damage patterns. Accordingly, the extent of lipid-rich myelin debris visualised by Oil Red O staining was higher in A2aR^+/+^ animals (Fig. 3f). In light of these findings, we quantified myelin pathology by Oil Red O staining in EAE animals treated with the A2aR-agonist CGS21680 (see therapeutic treatment paradigm, EAE course shown in Fig. 2b) and confirmed a similar pattern, characterised by increased myelin debris accumulation in the white matter (Additional file 1: Figure S5). No effects of CGS21680 could be observed on molecules related to macrophage/microglia functions, e.g. expression of iNOS or MHC class I (Additional file 1: Figure S6). Taken together, a similar effect on myelin damage patterns was detectable in two independent approaches, i.e. pharmacological and genetic modulation of A2aR.

### A2aR is expressed in macrophages/microglia within CNS lesions and inhibits macrophage and microglia function

**Fig. 4:**
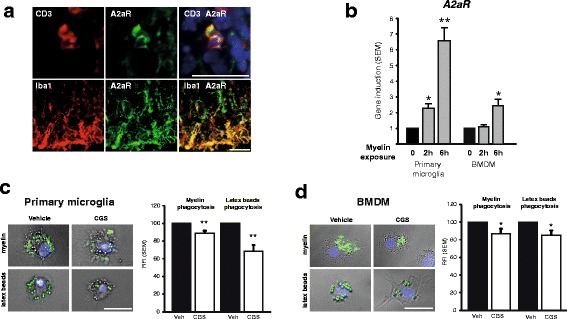
Analysis of A2aR-dependent modulation of phagocytosing cells. **a** CD3^+^ T cells and Iba1^+^ microglia/macrophages (*red*) co-stain for A2aR (*green*; in right overlay picture, *yellow*). Scale bars = 25 μm. **b** qPCR analysis of A2aR gene induction 2 and 6 h after addition of myelin to primary microglia and bone marrow-derived macrophages (*BMDM*) cultures. **c**, **d** Capacity of phagocytosis of labelled myelin lipoproteins and labelled carboxylate-modified polystyrene beads in primary microglia **(c)** and BMDM **(d)**, treated with vehicle or CGS21680. *Bar plots* depict pooled data from 4–8 experiments. Representative images of microglia and macrophages with phagocytosed myelin and beads. Scale bars = 25 μm. *p < 0.05, **p < 0.01

In order to identify the cells targeted by CGS21680 in the CNS, we performed an immunohistochemistry analysis of A2aR in inflamed CNS. Additionally to CD3^+^ T cells in EAE lesions, we observed the expression of A2aR on cells staining positive for Iba1, i.e. microglia and macrophages (Fig. 4a; see also Additional file 1: Figure S7 for A2aR/GFAP staining), which is in line with other findings in mouse inflammatory brain [34]. In light of recent insight into the role of infiltrating macrophages, as well as resident microglia in myelin pathology in EAE [42], we considered a role of these cells in our observations and we aimed to analyse A2aR-driven effects on these cells. Both cultured primary microglia and BMDM were shown to express A2aR (see Additional file 1: Figure S8). To analyse whether myelin debris may activate A2aR, we exposed cultured primary microglia cells and BMDM to purified mouse myelin. Indeed, treatment with myelin rapidly upregulated A2aR in both cell types (Fig. 4b). As phagocytic clearance of debris is an important prerequisite for remyelination [43–45], we investigated the impact of A2aR stimulation on phagocytosis in primary microglia (Fig. 4c) and macrophages (Fig. 4d), as well as migration capacity of these cells (Additional file 1: Figure S9). Agonistic A2aR stimulation had an inhibitory effect on phagocytosis (Fig. 4c, d). Furthermore, CGS21680 had an inhibitory effect on microglial and BMDM migration (Additional file 1: Figure S9).Taken together, these data suggest that induction and activation of A2aR on microglia and macrophages may inhibit phagocytic activity.

## Discussion

Adenosine has emerged as a sensor and regulator of tissue injury, particularly through A2aR-mediated modulation of inflammatory processes in different pathologies including autoimmune-mediated damage. Moreover, adenosine is an accepted signaling molecule within the CNS, and its metabolism is linked to energy homeostasis and reflects metabolic tissue alterations. In light of these reported effects of adenosine, we investigated the contribution of this purine metabolite to chronic autoimmune neuroinflammation.

We here report a profound and complex involvement of adenosine A2a receptor signaling in EAE, an animal model of multiple sclerosis. We show that A2aR is upregulated in the CNS tissue during experimental autoimmune attack, in line with a recent PET study on A2aR upregulation in the CNS of MS patients [46]. We next showed that pharmacological activation of A2aR in EAE using the specific agonist CGS21680 led to prevention of the disease when used in early disease stage. We show that T cells are involved in CGS21680-mediated early disease amelioration as their ex vivo proliferation was diminished; donor T cells from CGS21680-treated animals illicited less strong disease in recipient mice and their migratory capacity was reduced upon A2aR activation. Of note, this is in line with a human study showing that A2aR is upregulated in MS circulating lymphocytes and is having an anti-inflammatory effect in vitro [47]. It is also in line with previous EAE studies using A2aR-deficient mice showing that bone marrow-derived cells are responsible for an early disease exacerbation in A2aR-knockout mice [25, 26]. Using the same knockout model, we could confirm and extend these findings as genetic ablation of the receptor lead to a stronger activation of the immune system, reflected by a marked Th17 shift. Importantly, we were able to correlate this pro-inflammatory phenotype with an induction of GM-CSF-producing T helper cells, suggested to play a major role in the development of EAE [40]. Thus, if considered in isolation, these results may implicate pharmacological A2aR activation as a simple and promising anti-inflammatory therapeutic strategy in EAE and multiple sclerosis.

Paradoxically, the A2aR antagonist SCH58261 had also been shown to confer protection from EAE [24]. Indeed, our data implicate a much higher degree of complexity of A2aR signaling during chronic neuroinflammation as (1) the late use of the A2aR agonist CGS21680 confered protection from the disease (i.e. completely opposite effect of the same drug used in early stages) and (2) the clinical difference between KO and WT disappeared after an extended follow-up. These findings led us to hypothesise that the relevance of A2aR for the pathogenesis of chronic autoimmune neuroinflammation may depend on the time point or the compartment, i.e. the systemic immune response vs. the central nervous system.

Regarding this late-stage A2aR effect, our histological analysis of A2aR-deficient mice revealed a remarkable feature: whereas in late-stage EAE, the number of inflammatory foci was the same as in wild type, the number of foci with marked amount of myelin debris was higher in wild type. Recent evidence based on a unique transgenic mouse model suggests a role for macrophages and microglia in regulating demyelination and myelin debris clearance in EAE [42]. We found that A2aR was expressed on Iba1-positive cells in EAE lesions (i.e. microglia and macrophages) and in vitro myelin exposure led to an upregulation of the receptor. Furthermore, we could show that A2aR activation inhibited myelin uptake by both microglia and macrophages in vitro. It can be speculated that this effect provides a mechanism for our in vivo finding of decreased accumulation of myelin debris in the absence of A2aR signaling. However, other functions of macrophages and microglia could be involved in these processes, as we could also show an effect of A2aR on migration in both cell types. A prominent role of microglia (as compared to peripheral macrophages) could be inferred by the recent study of Yamasaki et al. [42] implicating macrophages to be rather involved in demyelination and microglia in myelin debris clearance. Our in vitro data, however, fall short of distinguishing these effects. Further in vivo experiments investigating microglia and macrophages separately, as well as other immune cells, would be required.

The data shown here may provide more insight into the controversial findings on the role of A2aR in neuroinflammation. For example, activation of A2aR downmodulates neuroinflammation and prevents tissue damage in models of intracerebral haemorrhage [48], kainate-induced hippocampal excitotoxicity [49], brain ischemia [50] or spinal cord injury [18, 19]. On the other hand, a number of reports have shown a beneficial effect of pharmacological and genetic inactivation of A2aR, for example, in brain ischemia [13], neuroinflammation-induced hippocampal damage [51] or cortical concussion [17]. In all these works, inflammatory pathways have been found to be crucial to the phenotypes. These seemingly paradox findings may ultimately be merged by the notion that A2aR derived from cells of different compartments confers differential effects. Indeed, reports investigating, e.g. ischemic stroke [15] or spinal cord injury [18], found that A2aR activation on bone marrow-derived cells rather confer neuroprotection and A2aR activation on non-bone marrow-derived cells (i.e. brain tissue cells) rather confer neurodegeneration. Though falling short of direct evidence, our data of early- and late-phase A2aR activation could be interpreted similarly, given that EAE is a disease that starts with the formation of a peripheral immune response in the early phase and after the breach of the blood-brain barrier evolves into a vastly complex interplay of the immune system with the cells of the CNS. Such dual roles of CNS inflammation confering injury as well as protection have reached a considerable amount of attention recently [52, 53]. Our data implicate A2aR as yet another Janus-faced molecule crucially involved in CNS autoimmunity.

## Conclusions

Taken together, we demonstrate a crucial but complex involvement of A2aR in the pathogenesis of chronic neuroinflammation. These findings extend our current understanding of adenosine-mediated regulation of immunological and neurobiological processes during myelin-specific autoimmunity.

## Additional file

Additional file 1:
**Supplementary Figures S1 to S9.**

## Abbreviations

A2aR: adenosine receptor A2a
APC: antigen-presenting cells
ATP: adenosine triphosphate
CFA: complete Freund’s adjuvant
CNS: central nervous system
EAE: experimental autoimmune encephalomyelitis
GM-CSF: granulocyte macrophage colony-stimulating factor
IFN: interferon
IL: interleukin
M-CSF: macrophage colony-stimulating factor
MOG: myelin oligodendrocyte glycoprotein
MS: multiple sclerosis
NECA: 5′-*n*-ethylcarboxamidoadenosine
PLP: proteolipid protein
PTX: pertussis toxin
RAG: recombination-activating gene
TCR: T cell receptor
