# Supplementary material for: Dual roles of the adenosine A2a receptor in autoimmune neuroinflammation
Source: J Neuroinflammation. 2016 Feb 26;13:48. doi: 10.1186/s12974-016-0512-z (PMC4768407; doi:10.1186/s12974-016-0512-z)
Supplement: Additional file 1: — Supplementary Figures S1 to S9. [file 12974_2016_512_MOESM1_ESM.pdf]

# Figure S1

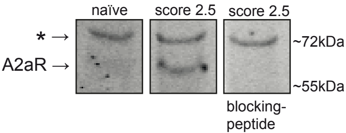


Demonstration of specificity of A2aR antibody by use of a blocking peptide.

## Figure S2

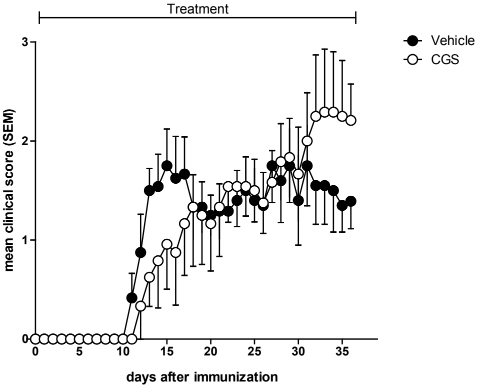


Treatment of wildtype C57BL/6 animals with daily i.p. injections of CGS21680 (0.1mg/kg) beginning at the day of immunization throughout the whole disease course leads to a disease amelioration in early EAE and a detrimental effect on disease severity in late EAE (n=6).

# Figure S3

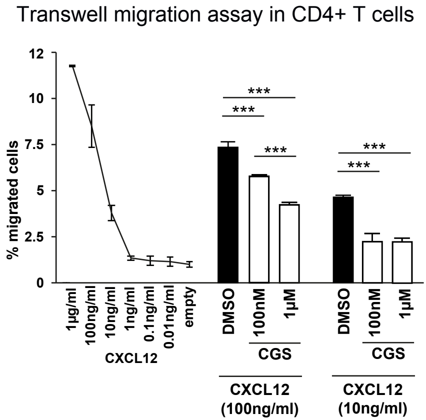


Magnetic bead-isolated CD4+ mouse cells were tested for transwell migration towards a CXCL12 gradient. The left graph shows migration to a gradient dilution, the middle group of columns shows a dose-dependent CGS21680 effect on migration towards a 100ng/ml CXCL12-gradient, the right group of columns shows an effect of CGS21680 on migration towards a 10ng/ml CXCL12-gradient. Depicted is one representative experiment.

# Figure S4

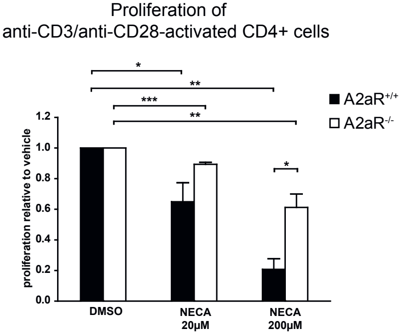


MACS®-sorted A2aR-/- or littermate wildtype CD4+ T cells were stimulated with α-CD3 (1µ/ml) and α-CD28 (1µg/ml) for 72h in the presence or absence of NECA, a synthetic analog of adenosine. Cells were tested for proliferation by 3H-thymidine assay (see methods).

# Figure S5

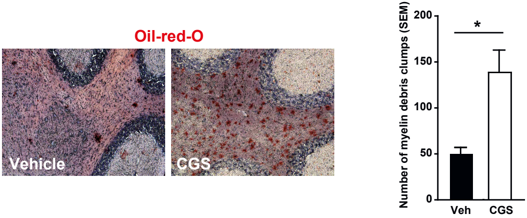


Oil-red-O/hematoxylin stainings in brain slices from mice treated with the A2aR-agonist CGS21680 after onset of disease (i.e. day 12 after immunization, therapeutic paradigm, see EAE shown in Figure 2B). Myelin debris appears to form clumps (red in representative images). Quantification was performed by counting the number of such myelin debris clumps per section in cerebellar white matter (3 sections per animal, n=4 animals). * $p<0.05$.

# Figure S6

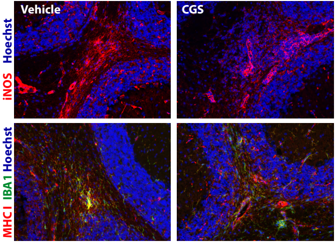


Immunohistochemical analysis of iNOS and MHC class I expression in vehicle and CGS treated EAE animals. Representative images from n=4 animals. Upper panel: red=iNOS, blue=Hoechst. Lower panel: red=MHC class I, green=Iba1, blue=Hoechst.

# Figure S7

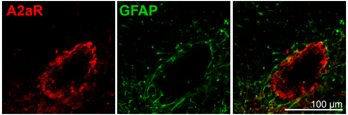


Immunohistochemical analysis of astrocytes for A2aR expression in EAE lesions.
Red=A2aR, green=GFAP.

# Figure S8

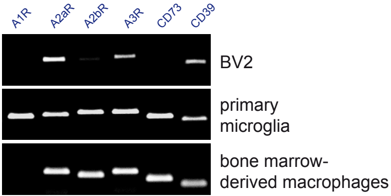


Expression of genes of the extracellular adenosine system in cultured microglial cells (BV2 and primary microglia) and bone marrow-derived macrophages.
Agarose gel electrophoresis of PCR products.

# Figure S9

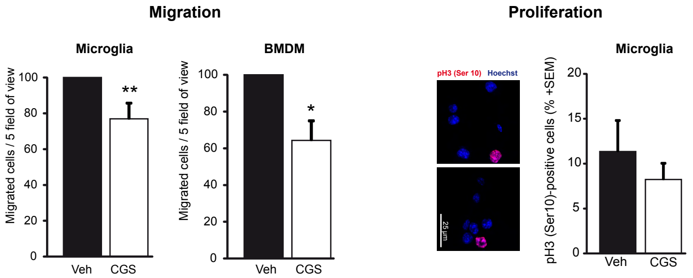


Effect of A2aR stimulation on migration and proliferation capacity *in vitro*.
